# Discovering molecules and plants with potential activity against gastric cancer: an *in silico* ensemble-based modeling analysis

**DOI:** 10.3389/fbinf.2025.1642039

**Published:** 2025-09-30

**Authors:** Micaela Villacrés, Alec Avila, Karina Jimenes-Vargas, António Machado, José M. Alvarez-Suarez, Eduardo Tejera

**Affiliations:** 1 Clínica San Cayetano, Quito, Ecuador; 2 Bio-Cheminformatics Research Group, Universidad de Las Américas, Quito, Ecuador; 3 Departament of Computer Science and Information Technologies, Faculty of Computer Science, Universidade da Coruña, Campus Elviña s/n, A Coruña, Spain; 4 Departamento de Biologia, Centro de Biotecnologia dos Açores (CBA), Faculdade de Ciências e Tecnologia, Universidade dos Açores, Ponta Delgada, Portugal; 5 Laboratorio de Bacteriología, Colegio de Ciencias Biológicas y Ambientales COCIBA, Instituto de Microbiología, Universidad San Francisco de Quito USFQ, Quito, Ecuador; 6 Laboratorio de Investigación en Ingeniería en Alimentos (LabInAli), Departamento de Ingeniería en Alimentos, Colegio de Ciencias e Ingenierías, Universidad San Francisco de Quito (USFQ), Quito, Ecuador; 7 Laboratorio de Bioexploración, Colegio de Ciencias Biológicas y Ambientales, Universidad San Francisco de Quito (USFQ), Quito, Ecuador; 8 Facultad de Ingeniería y Ciencias Aplicadas, Universidad de Las Américas, Quito, Ecuador

**Keywords:** gastric cancer prevention, plant-derived compounds, *in silico* screening, compound discovery, bioactive plant species, secondary metabolites

## Abstract

**Background:**

Gastric cancer (GC) remains a major global health burden despite advances in diagnosis and treatment. In recent years, natural products have gained increasing attention as promising sources of anticancer agents, including GC.

**Methods:**

In this study, we applied an *in silico* ensemble-based modeling strategy to predict compounds with potential inhibitory effects against four GC-related cell lines: AGS, NCI-N87, BGC-823, and SNU-16. Individual predictive models were developed using several algorithms and further integrated into two consensus ensemble multi-objective models. A comprehensive database of over 100,000 natural compounds from 21,665 plant species, was screened for validation and to identify potential molecular candidates.

**Results:**

The ensemble models demonstrated a 12–15-fold improvement in identifying active molecules compared to random selection. A total of 340 molecules were prioritized, many belonging to bioactive classes such as taxane diterpenoids, flavonoids, isoflavonoids, phloroglucinols, and tryptophan alkaloids. Known anticancer compounds, including paclitaxel, orsaponin (OSW-1), glycybenzofuran, and glyurallin A, were successfully retrieved, reinforcing the validity of the approach. Species from the genera *Taxus*, *Glycyrrhiza*, *Elaphoglossum*, and *Seseli* emerged as particularly relevant sources of bioactive candidates.

**Conclusion:**

While some genera, such as *Taxus* and *Glycyrrhiza*, have well-documented anticancer properties, others, including *Elaphoglossum* and *Seseli*, require further experimental validation. These findings highlight the potential of combining multi-objectives ensemble modeling with natural product databases to discover novel phytochemicals relevant to GC treatment.

## 1 Background

Despite significant advances in medicine, gastric cancer remains a major global public health challenge, characterized by a dynamic historical evolution in its incidence, diagnosis, and treatment. Traditionally, it has ranked among the leading causes of cancer-related mortality, particularly in regions with a high prevalence of *Helicobacter pylori* infection and unhealthy dietary patterns. During the 20th century, the incidence of gastric cancer markedly declined in developed countries, primarily due to improvements in hygiene, widespread use of food refrigeration, and reduced consumption of salted and smoked foods (Kang et al., 2024). However, it remains a substantial cause of cancer-related deaths worldwide, with a heterogeneous geographical distribution. East Asia, Latin America, and Eastern Europe report the highest incidence rates, whereas North America and Western Europe have experienced a continuous downward trend (Mithany et al., 2024). In 2020, more than 1 million new cases of gastric cancer were diagnosed globally, accompanied by approximately 769,000 deaths, underscoring the persistent magnitude of this disease (Sung et al., 2021). Notably, the epidemiological profile of gastric cancer has undergone a significant shift in recent decades, with an increasing incidence observed among younger populations. This trend has prompted a reevaluation of preventive strategies and emphasizes the critical need for early-life interventions targeting modifiable risk factors (Kang et al., 2024). Several determinants of gastric cancer have been well-established. Infection with *H. pylori* remains the most prominent biological risk factor, often acting synergistically with behavioral and environmental influences (Poorolajal et al., 2020). Socioeconomic disparities further modulate the burden of disease. Lower educational attainment and limited access to healthcare services are associated with unhealthy lifestyles and delayed diagnosis, ultimately impacting survival outcomes (Alicandro et al., 2022). These complex, interrelated factors highlight the necessity for comprehensive, multidisciplinary approaches to the prevention, early detection, and management of gastric cancer globally.

In the treatment of gastric cancer, various chemotherapeutic agents have demonstrated significant efficacy in both *in vitro* cellular model systems and preclinical studies. Capecitabine, a prodrug of 5-fluorouracil (5-FU), has been shown to inhibit cell proliferation and angiogenesis in experimental models using BGC-823 cells, improving survival outcomes with low toxicity (Yuan et al., 2015). Similarly, docetaxel, an agent that disrupts microtubule polymerization, has proven effective by inducing G_2_/M phase cell cycle arrest in AGS cells and exhibiting antiangiogenic and synergistic effects when combined with compounds such as gambogic acid in BGC-823 cells (Grabarska et al., 2023). Additional chemotherapeutic agents and alternative treatment strategies for gastric cancer have been extensively reviewed (Sexton et al., 2020; Guan et al., 2023). In parallel with the search for effective chemotherapeutic agents, increasing attention has been given to natural compounds. Curcumin, the principal bioactive component of turmeric, has demonstrated notable anti-inflammatory and antiproliferative properties relevant to the prevention and treatment of gastric cancer (Zhang et al., 2022). Various formulations of curcumin are currently being evaluated in clinical trials, such as NCT02782949, further supporting its potential application in gastric cancer management (Warias et al., 2024). Other natural molecules, including resveratrol, quercetin, and piceatannol, found in a variety of plant-derived products, have also shown the ability to modulate inflammatory processes and oncogenic pathways involved in gastric cancer progression (Zhao et al., 2023; Warias et al., 2024).

In the context of the discovery of novel natural products, *in silico* predictive modeling has emerged as a powerful methodology for identifying bioactive compounds. This approach employs computational tools to predict interactions between natural molecules and target proteins implicated in carcinogenesis, thereby accelerating the identification of novel therapeutic candidates (Liu et al., 2024a). Predictive modeling has been particularly instrumental in uncovering anticancer agents derived from food sources, notably polyphenols, which exhibit antioxidant and anti-inflammatory properties capable of reducing gastric cancer risk (Zheng et al., 2024). Computational strategies have been used to predict new drug targets, such as the epidermal growth factor receptor (EGFR) (Mashima et al., 2019), and to identify natural compounds, such as coumarin derivatives, capable of interacting with BCL2 and inducing apoptosis in gastric cancer cells (Perumalsamy et al., 2018). Moreover, the integration of network pharmacology approaches has enabled the identification of bioactive molecules like dehydroxy-isocalamendiol and spathulenol, which bind to critical cancer-related proteins, further highlighting their therapeutic potential (Pradhan et al., 2024). However, despite these advances, few studies have focused on large-scale screening of natural product libraries using phenotypic models, (Dai et al., 2016; Jin et al., 2020), as opposed to traditional target-centered modeling strategies (Jalali et al., 2023). Expanding the use of phenotypic screening could enhance the discovery of multifunctional compounds with broader mechanisms of action against gastric cancer. In this context, the present *in silico* study aims to identify effective molecules against gastric cancer (GC) cell lines, specifically AGS, NCI-N87, SNU-16, and BGC-823. Building upon our previous work (Perez-Castillo et al., 2018), we employed a consensus approach based on ensemble modeling, where individual predictive models were constructed and subsequently integrated to generate a final consensus probability for each compound. This methodology was applied to screen more than 100,000 molecules derived from 21,665 plant species. The ultimate objective is to pinpoint potential natural sources and plant species that could serve as promising candidates for further research in drug discovery and drug design collectively targeting GC cell lines.

## 2 Materials and methods

**FIGURE 1:**
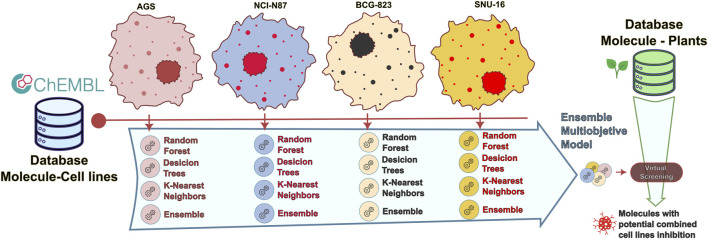
Flow diagram of the presented methodology.

A full schematic representation of the methodology is presented in Figure 1. This representation will be described in detail across this section.

### 2.1 Database’s description and curation

Four gastric cancer-related cell lines were selected for modeling: AGS (ChEMBL 3308078), NCI-N87 (ChEMBL 3307326), BGC-823 (ChEMBL 3307635), and SNU-16 (ChEMBL 3307273). All compounds with reported IC_50_ values were retrieved from the ChEMBL database, version 35 (Zdrazil et al., 2024). The data curation process followed a strategy similar to that described in previous studies (Perez-Castillo et al., 2018; Tejera et al., 2021). An IC_50_ threshold of 10 µM was employed to classify compounds as active (<10 µM) or inactive (>10 µM). When a molecule had multiple IC_50_ values reported for the same cell line across different studies, it was included only if all reports consistently classified it in the same activity class (i.e., all experiments agreed on its active or inactive status). If this criterion was not met, the compound–cell line pair was excluded. Additionally, compounds evaluated in more than two cell lines were excluded from the training sets and instead reserved for virtual screening purposes.

### 2.2 Modeling strategies and predictions

All molecules were described using ECFP4 fingerprints (1,024 bits) computed with RDKit (RDKit, 2018). Only ECFP4 description was used in this work. We had used this type of description previously in molecule-cell lines interaction across several cell lines (Tejera et al., 2019). However, we agree that it is not the only option available. Given that class imbalance is commonly observed, typically favoring either the inactive or active class, data balancing was performed through data reduction by applying a clustering algorithm to the majority class, following a strategy similar to that used in previous work (Tejera et al., 2021). Specifically, all molecules in the majority class (represented by their ECFP4 fingerprints) were clustered using the k-means algorithm (KMeans function from *sklearn. cluster*), incrementally increasing the number of clusters from 2 up to the number of compounds in the minority class. For each clustering step, the silhouette score was calculated (using the *silhouette*_score function from *sklearn. metrics*), and the number of clusters yielding the highest silhouette score was selected as optimal. Once the optimal number of clusters was determined, a proportional number of compounds was randomly selected from each cluster to match the size of the minority class. The final balanced datasets for each cell line are presented in Supplementary Material S1.

After balancing the data, a random split was performed for each cell line dataset into training, test, and external sets, following a 60%-20%-20% ratio. Prior to modeling, variable reduction was applied by removing all descriptors with a variance lower than 0.05 within the training subset. An important aspect of model evaluation (for both test and external sets) is the consideration of the applicability domain. To define this domain, a principal component analysis (PCA) was conducted on the training subset, extracting the principal components that together explained more than 90% of the cumulative variance. The maximum Euclidean distance between individual compounds and the centroid (computed using the selected principal components) was used to define the applicability domain. Any compound whose distance exceeded this maximum value was excluded from further analysis. The reason to use Euclidian distance is that the principal components are normalized numerically continuing description and it is fast, intuitive and simple to compute. In previous works we used the Tanimoto distance directly on the ECFP4 fingerprint (Jimenes-Vargas et al., 2024). This approach could be robust but computing the similarity matrix is computationally expensive over large datasets.

We evaluated four modeling strategies: random forest (RF), decision trees (DTREE), k-nearest neighbors (KNN), and an ensemble modeling approach combining models derived from RF, DTREE, and KNN. In the case of RF and DTREE we used Gini impurity to measure the quality of the split. Regarding the maximum depth of the tree, we initially explored several values and decided to restrain to 100 for RF. In the case of DTREE all nodes are expanded until all leaves contain less than 2 samples. These parameters were not modified further in the analysis or optimization. Additionally, for variable selection in the RF, DTREE, and KNN models, a genetic algorithm was employed (Perez-Castillo et al., 2018; Tejera et al., 2021). The genetic algorithm was performed with an initial population of 1,000 individuals and was executed over 5,000 generations. To ensure model simplicity and prevent overfitting, the number of variables selected in each generated model was restricted to between 4 and 25. The balanced classification rate (BCR) was used as the fitness function for the genetic algorithm (Equation 1) (Perez-Castillo et al., 2018).

$$BCR=\frac{Se+Sp}{2}\left(1-\left|Se-Sp\right|\right)$$ (1)

where *Se* and *Sp* are the sensitivity and specificity respectively. For each model, we computed: *BRC*, *Se*, *Sp*, F1-score, and accuracy for the test and external validation.

### 2.3 Ensemble modeling and virtual screening

For ensemble modeling, an initial population of 200 individual models was generated, each fulfilling the following criteria: (i) each model randomly used one of the RF, DTREE, or KNN algorithms; (ii) each model included between 4 and 25 variables; and (iii) each model achieved a BCR higher than 0.65 when evaluated on the test set. Based on these individual models, an initial ensemble population of 1,000 random combinations was created, with each ensemble comprising between 2 and 20 models. This initial ensemble population was further optimized using a genetic algorithm aimed at maximizing the BCR metric over 5,000 generations. In the final ensemble models, the mean probability of the individual models was used as the aggregation function to compute the final prediction.

For the virtual screening phase, several considerations must be addressed. The primary objective of the final models is to evaluate the probability that a compound exhibits anticancer activity, specifically against gastric cancer. Thus, the main challenge lies in accurately ranking compounds according to this criterion. However, defining anticancer activity based solely on cell line data presents difficulties, as (a) we are evaluating effects across four different gastric cancer cell lines, and (b) most compounds do not have activity data reported for all four cell lines, indeed, only two compounds in the ChEMBL database were found to have information across all four. To assess the performance of the combined cell line models in identifying potentially useful molecules, a virtual screening dataset was constructed. This dataset included: (i) 51 compounds from ChEMBL with reported activity against at least three of the four cell lines. Of these, 25 compounds were classified as active (active in at least two cell lines), while the remaining compounds were classified as inactive (active in only one or none of the cell lines). Additionally, (ii) compounds from the Genomics of Drug Sensitivity in Cancer (GDSC) database (Yang et al., 2012) were retrieved, specifically those evaluated across AGS, NCI-N87, and SNU-16 cell lines (BGC-823 data were not available in GDSC). A total of 125 compounds were identified and classified as active or inactive based on a z-score threshold of −1.5. Following a similar approach as with the ChEMBL dataset, 62 compounds were classified as active (active in at least two cell lines), and the remainder as inactive.

Finally, we also consulted the National Cancer Institute database (National Cancer Institute, 2025) and included four of the 22 small-molecule drugs currently used in the treatment of GC: capecitabine, docetaxel, doxorubicin, and mitomycin. Fluorouracil was not included, as it is already present in the GDSC database and was found to be inactive against the three gastric cancer cell lines evaluated. With this additional information, a total of 178 molecules were compiled for the virtual screening dataset, of which 86 were labeled as “active,” indicating a higher likelihood of exhibiting anticancer activity.

One limitation of the constructed dataset is its relatively small size, which restricts the evaluation of enrichment metrics for virtual screening. For a robust calculation of virtual screening performance, a larger number of inactive (negative) molecules is required. As previously described (Perez-Castillo et al., 2018), decoy molecules were generated from the 86 active compounds. To this end, the DUD-E web service (Mysinger et al., 2012) was utilized, resulting in the generation of 4,357 decoy molecules, leading to a final dataset comprising 4,535 compounds. The early recognition metrics used to evaluate the models’ performance in virtual screening are defined in Equations 2, 3 (Truchon and Bayly, 2007).

If ${RIE}_{\min}=\frac{1-e^{-\alpha Ra}}{Ra\left(1-e^{\alpha }\right)}$ and ${RIE}_{\max}=\frac{1-e^{-\alpha Ra}}{Ra\left(1-e^{-\alpha }\right)}$ , then we can define BEDROC as:

$$BEDROC=\frac{RIE-{RIE}_{\min}}{{RIE}_{\max}-{RIE}_{\min}}$$ (2)

In these equations, *Ra = n/N*, where *n* is the number of active compounds and *N* is the total number of molecules. The *α*-value corresponds to the portion of the ranked dataset where the recovery of active compounds is evaluated, representing early recognition performance. Mathematically, the *α* -value is associated with a fraction 0<χ≤1, indicating the segment of the ranked list within which the active compounds are retrieved. This fraction is also necessary for computing the enrichment factor (EF), which is defined as:

$$EF=\frac{\sum_{i=1}^{n}\delta_{i}}{\chi^{n}},\text{where }\delta_{i}=\left\{\begin{array}{cc} 1 & r_{i}\leq \chi N\\ 0 & r_{i}>\chi N \end{array}\right.$$ (3)

Here, *N* represents the total number of compounds in the virtual screening list. Higher values of *α* correspond to smaller fractions of the ranked list used to retrieve active compounds, emphasizing early recognition. The computation of *α* can be adjusted depending on specific evaluation goals (Truchon and Bayly, 2007). For example, to evaluate enrichment within the top 1% of the ordered list while aiming for this fraction to contribute approximately 80% of the overall enrichment, an α-value of 160.9 is used. In our study, enrichment metrics were computed under different α-value and χ conditions to thoroughly assess model performance.

### 2.4 Compound-plants databases curation

To create a compound–plant database, information was integrated from FOODB (Foodb, 2025), COCONUT (Sorokina et al., 2021), and LOTUS (Rutz et al., 2022) databases. COCONUT and LOTUS are specialized in natural products. In both the COCONUT and FOODB databases, full taxonomic identification of the source species is not always available. FOODB includes entries corresponding not to specific species but rather to processed or mixed foods (e.g., popcorn, cheese, milk), which were excluded from this analysis. To standardize species names, we used the National Center for Biotechnology Information (NCBI) taxonomy database (*fullnamelineage.dmp*), considering only plant species (Viridiplantae) that could be matched in NCBI records. Regarding compound curation, the RDKit package for Python was employed to perform the following operations: (1) removal of chiral information, (2) generation of InChIKeys, and (3) molecule sanitization. The removal of chiral information was based on two key considerations: (i) chiral descriptors are inconsistently reported across databases, either because the absolute configuration is unknown or because compounds exist as racemic mixtures, and (ii) the predictive models developed in this study do not account for chirality. Thus, treating enantiomers as distinct entities could artificially inflate the number of predicted active compounds. Consequently, enantiomers were considered duplicate entries when found within the same plant species. After filtering and cleaning the data, the final database included 21,665 plant species, 105,938 unique compounds, and 2,251,567 compound–species associations.

## 3 Results

Following the curation and balancing procedures, we characterized the datasets based on their active and inactive compound distributions. The final datasets (provided in Supplementary Material S1, SM1) comprised SNU-16 (n = 210; 100 active, 110 inactive), NCI-N87 (n = 247; 130 active, 117 inactive), BGC-823 (n = 1,565; 746 active, 819 inactive), and AGS (n = 791; 396 active, 395 inactive).

### 3.1 Predictive models and virtual screening

The performance of each applied algorithm is summarized in Table 1. We report both the best model identified within the final genetic algorithm populations and the mean performance metrics across the entire final population. Complete performance data for all models and ROC curves representations are provided in Supplementary Table S2.1 and Supplementary Figure S2.1 of Supplementary Material S2(SM2).

**TABLE 1:** Performance metrics of the different modeling strategies, including ensemble approaches.

Cell Lines	Models	GA results		Best model				
		Test		Test		External		
		Mean ACC	Mean BCR	ACC	BCR	ACC	BCR	NV^a^
SNU-16	RF	0.825	0.806	0.857	0.776	0.810	0.793	19
	DTREE	0.796	0.778	0.786	0.748	0.857	0.825	26
	KNN	0.834	0.814	0.810	0.810	0.810	0.793	17
	ENSEMBLES	0.861	0.857	0.857	0.857	0.762	0.759	6
NCI-N87	RF	0.847	0.822	0.816	0.802	0.816	0.802	23
	DTREE	0.840	0.816	0.816	0.802	0.837	0.819	36
	KNN	0.812	0.787	0.816	0.802	0.837	0.819	25
	ENSEMBLES	0.929	0.915	0.918	0.909	0.857	0.838	11
BGC-827	RF	0.688	0.667	0.703	0.687	0.709	0.705	17
	DTREE	0.786	0.774	0.789	0.764	0.802	0.795	42
	KNN	0.788	0.775	0.792	0.782	0.805	0.782	33
	ENSEMBLES	0.858	0.851	0.863	0.853	0.815	0.809	15
AGS	RF	0.749	0.736	0.759	0.740	0.772	0.772	25
	DTREE	0.768	0.753	0.766	0.737	0.778	0.769	23
	KNN	0.764	0.749	0.753	0.744	0.778	0.769	36
	ENSEMBLES	0.856	0.845	0.880	0.869	0.823	0.823	8

^a^ NV: Number of variables included in each model. For ensembles, this number indicates the number of models integrated into the final ensemble.

**FIGURE 2:**
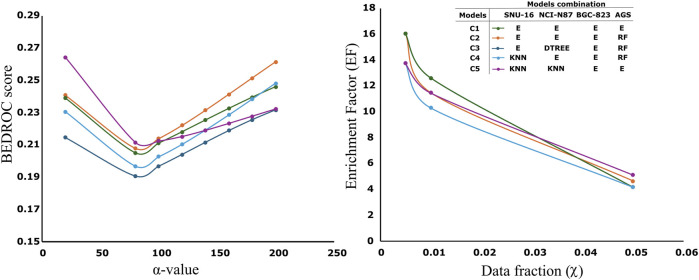
Left: Variation of the BEDROC values across different α-values for various model combinations. Right: Variation of the enrichment factor (EF) across different top fractions (χ) for the same model combinations. Each line represents a model combination from Table 1 for each cell line. The notation “E” denotes the ensemble model corresponding to each cell line, as described in Table 1.

The selection of the best model from all models after genetic algorithm optimization is supervised. We consider a high BCR value in the test and external partitions. However, we also consider similar values between the mean BCR value from the genetic algorithm population and the BCR obtained in the external partition. This similarity is extended to other metrics like sensitivity, specificity and F1-score that can be consulted in SM2. We observe that, in some cases, ensemble models perform slightly better than the other models. For instance, this is the case for the AGS and BGC-827 cell lines. However, for the NCI-N87 cell line, decision tree models appear to outperform ensembles, while in the SNU-16 cell line, random forest and KNN models also seem to perform better. It is important to notice that we did not use cross-validation. The models are trained using the “training” partition and the fitness functions during model evolution are obtained by evaluation in the “test” group. The final models are evaluated in the “external” partition which is not used at any moment of the training or variable selection (Tropsha, 2010; Castillo-González et al., 2015). We can notice that the performance metrics are quite similar across the test group (presented as the average across the entire population), and the external partition which is a good indicator of the model’s stability and generalization. All compounds used for virtual screening (n = 4,535) were evaluated with all models. To select the best combination of models providing the highest initial enrichment, we evaluated all possible model combinations (256 combinations) and computed the BEDROC score and enrichment factor (EF) across several initial enrichment fractions. The best combinations are presented in Figure 2 (individual model values are detailed in Supplementary Table S2.2; Supplementary Material S2).

Higher α-values assign greater weight to the BEDROC score in the early enrichment regions of the ranked dataset. Values around *α* = 160 correspond approximately to the top 1% fraction (0.01 in Figure 2, Right) (Zhang et al., 2017). Notably, the EF results for the combinations E, DTREE, E, RF (dark blue line) and E, E, E, E (green line) are identical. In the screening procedure we desire models capable of correctly identifying (or retrieving) most of the active molecules (it is what BEDROC and EF quantify) in the minimal portion of the ranked list (it is computed by the fraction and/or the *α* -value). Analysis of the profiles in Figure 2 suggests that the two best-performing combinations are: C1) E, E, E, E (green line) and C2) E, E, E, RF (orange line). C2 exhibits a superior BEDROC score at α = 160 and across higher α-values, while C1 achieves a higher EF. The enrichment factors (EF) of C1 and C2 in the top 0.5%–1% (χ = 0.005–0.01) of the ranked list are between 12 and 16, suggesting suggestion 12 to 16 times better enrichment that what we should expect from random. In our case, we had a total of 86 active molecules in a total of 4,535, so in the top 1% (45 molecules), we ranked 14 and around 7 in the top 22 (close to 0.5%) ranked molecules. The maximum average probabilities obtained across the entire virtual screening dataset for C1 and C2 were 0.651 and 0.641, respectively.

### 3.2 Molecules and plants sources

After database curation as described, a total of 105,938 unique SMILES were obtained. However, after applying the applicability domain filters of the models comprising C1 and C2, 104,408 SMILES remained. Among these, 6,736 molecules (6.45%) in C1 and 5,512 (5.28%) in C2 exhibited a predicted probability greater than 0.5. The maximum predicted probabilities for C1 and C2 were 0.681 and 0.626, respectively, consistent with the peak values observed in the virtual screening dataset. Interestingly, only 91 molecules had a probability >0.6 in C1, compared to just 23 in C2, with only 8 compounds shared between the two models. This discrepancy suggests that the C2 model is more restrictive. The number of shared molecules between C1 and C2 at probability thresholds >0.5, >0.55, and >0.6 were 3,778, 340, and 8, respectively. No compounds were shared above the 0.65 threshold, as only C1 identified three molecules at that level. The 340 shared molecules at the 0.55 threshold were classified using NPClassifier (Kim et al., 2021), and their chemical classes, as well as the associated C1 and C2 probabilities, are provided in Supplementary Material S3 (SM3).

**FIGURE 3:**
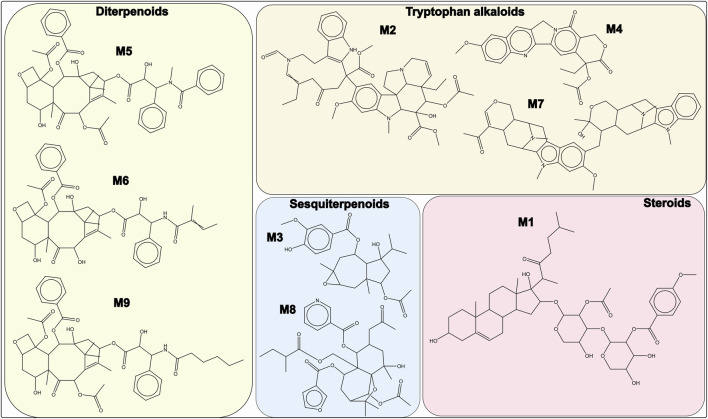
Molecules M1–M3 represent the top-ranked compounds in the C1 model (score >0.65). Molecules M1, M2, and M4–M9 are the eight compounds identified in both C1 and C2 models with scores >0.6. These represent the highest-scoring molecules across both models.

**FIGURE 4:**
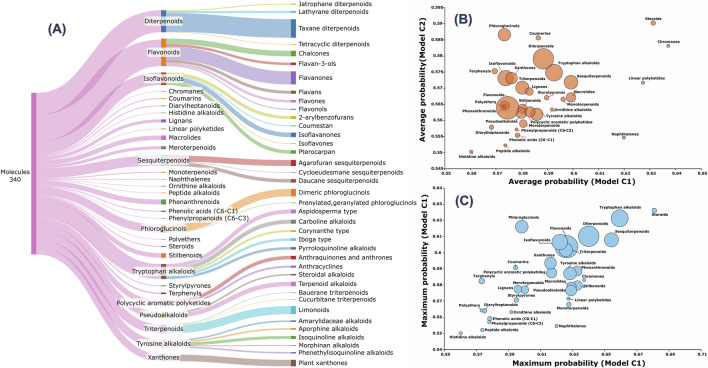
Analysis of molecular classes and subclasses. **(A)** Distribution of molecules by chemical class and subclass. **(B)** Average probabilities predicted by the C1 and C2 models for each chemical class. **(C)** Maximum probabilities predicted by the C1 and C2 models for each class. Bubble sizes in panels B and C represent are proportional to the number of compounds within each chemical class.

The top-ranked molecules (the top 3 from the C1 model and the 8 common molecules identified by both C1 and C2) are shown in Figure 3, along with their corresponding chemical classes. Molecule M3 is the only compound predicted by the C1 model with a score >0.65 that is not among the 8 shared molecules. Notably, M1 corresponds to orsaponin (PubChem CID: 72612554), M6 to 7-epi-10-Deacetyl Cephalomannine (PubChem CID: 72738999), and M9 to paclitaxel (PubChem CID: 23509308). The remaining molecules do not have common names or standardized notations in PubChem. It is also worth noting that M5 displays strong structural similarity to both M6 and M9. Flavonoids, isoflavonoids, di- and triterpenoids, sesquiterpenoids, and tryptophan-derived alkaloids represent the majority of the 340 molecules with combined probabilities greater than 0.55 (Figure 4A). Among the diterpenoids, taxane-type diterpenoids are the most abundant; this subclass includes compounds whose names are derived from the plant genus Taxus (to be discussed later). Within the flavonoid class, flavanones are the most represented subclass, followed by chalcones. In the isoflavonoid group, isoflavanones and pterocarpans are the most common. Pterocarpans are typically found in the Fabaceae family, while isoflavanones are broadly distributed, as are agarofuran and daucane (carotane-type) sesquiterpenoids.

The classes with the highest number of molecules, namely, flavonoids and diterpenoids, do not necessarily contain the compounds with the highest predicted probabilities (Figures 4B,C). The coefficient of determination (R^2^) between the predicted probabilities of the C1 and C2 models across the entire set of 104,408 molecules is 0.743. However, this value drops substantially to R^2^ = 0.108 when considering only the 340 shared molecules with a probability cutoff >0.55, suggesting that although the models are overall similar, they are specialized in distinct regions of chemical space. For instance, both models tend to assign higher probabilities to steroids and chromanes (Figure 4B), while phloroglucinols, coumarins, and diterpenoids are more highly ranked by the C2 model. In contrast, naphthalenes and linear polyketides are more strongly favored by the C1 model. Regarding maximum predicted probabilities, there is better consistency between C1 and C2 (Figure 4C), although phloroglucinols continue to be among the top-ranked classes in the C2 model. To identify the most relevant plant species, it is necessary to consider the number of potentially active compounds, their predicted probabilities, and the total number of compounds reported for each species. Some species are well-represented in the database, such as *Garcinia mangostana* and *Syzygium aromaticum*, while others are represented by only a few compounds, such as *Melicope durifolia* and *Turraea obtusifolia*. Out of a total of 21,665 species, only 1,045 (4.82%) contain at least one compound among the 340 molecules commonly identified by C1 and C2 models with a probability greater than 0.55. Moreover, only 215 species (0.99%) have two or more such compounds, and just 37 species (0.17%) have five or more. The average predicted probabilities from the C1 and C2 models, the number of identified active compounds, and the total number of compounds reported for each species are presented in SM3.

**FIGURE 5:**
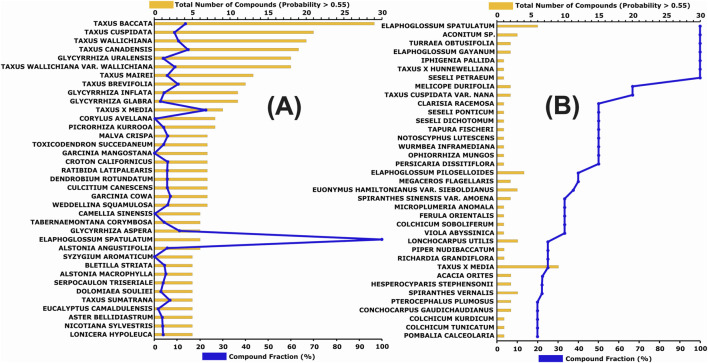
**(A)** Species with five or more active compounds, sorted by the total number of active compounds (orange bars) and the percentage of active compounds relative to the total number of molecules reported for the species (blue line). **(B)** Species sorted by the fraction of active compounds (blue line) and the corresponding number of active compounds (orange bars).

Several species from the genus *Taxus* (e.g., *Taxus baccata*, *Taxus cuspidata*) contain the highest number of predicted active compounds (Figure 5A), although the proportion of active compounds relative to the total number of molecules reported per species varies considerably (from 10% to 100%) (Figure 5B). In contrast, several species from the genus *Elaphoglossum* (*E. spatulatum*, *E. gayanum*, and *E. piloselloides*), despite having fewer compounds reported in the database (between 3 and 6), exhibit a high proportion (40–100%) of compounds with predicted probabilities >0.55.

Considering both absolute and relative representations, as well as the compound class results shown in Figure 4, the genera *Taxus*, *Elaphoglossum*, *Glycyrrhiza*, and *Seseli* appear particularly relevant for gastric cancer-related bioprospecting. In general, some chemical subclasses are distributed across various plant species, while others are more genus- or family-specific. For further analysis, all species from the aforementioned genera were grouped, resulting in 36, 20, 11, and 1 identified active compounds for *Taxus*, *Glycyrrhiza*, *Elaphoglossum*, and *Seseli*, respectively. Although several *Seseli* species appear in Figure 5B, they are all associated with a single compound (PubChem CID: 163026028), a tryptophan alkaloid. The dominant chemical classes identified within each genus were: 97.22% diterpenoids in *Taxus*, 85.00% isoflavonoids in *Glycyrrhiza*, 90.91% phloroglucinols in *Elaphoglossum*, and 100% tryptophan alkaloids in *Seseli*. This distribution highlights the presence of distinct bioactive scaffolds across different botanical lineages, underscoring their potential for targeted pharmacological exploration.

## 4 Discussion

In the individual models developed for each cell line, at least one modeling strategy achieved an accuracy greater than 0.8, although none surpassed 0.9. This trend was also observed in a previous study on the same cell lines (Perez-Castillo et al., 2018), except for one newly included cell line in the present work. In the previous work of (Perez-Castillo et al., 2018) we used a different fingerprint representation. Even though the objective is not focused on the analysis of the best chemical representation for this type of interactions, our results seem to indicate that ECFP4 description is slightly better than the description previously used. The ISIDA Fragmentor software (Varnek et al., 2008) was previously used to obtain 2D derived fingerprints descriptors that are quite different to the ECFP4. Even when the presented work is not directly comparable (i.e., different datasets) to our previous work, the performance metrics in the presented modelling are better. Our consensus models showed an accuracy range of 0.759–0.838 compared to 0.624–0.768 in the external dataset in our previous work (Perez-Castillo et al., 2018) while four and not three cell lines were used (we added the BGC-827). Several factors could explain this behavior including the increment of the compounds in the database. However, we should keep in mind that when modeling compound–cell line interactions, multiple mechanisms may underlie cell mortality and each mechanism could be potentially associated with distinct chemical spaces (i.e., targeting different molecular pathways or proteins). Moreover, the chemical space represented in the database for each cell line likely does not capture all these mechanisms uniformly. This hidden structure will increase the challenge for machine learning models to fully recognize and balance the internal chemical diversity. Notably, combining the models for different cell lines during virtual screening improved the chances of identifying active molecules by 12–15 times compared to random selection.

Despite the therapeutic advances achieved with chemotherapeutic agents such as capecitabine and docetaxel in the treatment of gastric cancer, significant clinical limitations persist, warranting the exploration of new pharmacological strategies. Treatment efficacy remains limited in patients with advanced-stage disease, and a high incidence of acquired resistance leads to early relapses and reduced overall survival (Liu et al., 2024b). Moreover, the cumulative toxicity associated with prolonged chemotherapy compromises quality of life, particularly in elderly patients or those with comorbidities (Rupp and Stengel, 2021). These challenges highlight the urgent need to investigate alternative or complementary therapies. Moreover, the research on natural products could also reveal new avenues on potential alternative mechanisms in gastric cancer inhibition or provide a synergic complement (Mao et al., 2020).

In the presented work, the goal of this virtual screening experiment was to evaluate the models’ ability to retrieve compounds exhibiting inhibitory effects across more than two gastric cancer cell lines. Rather than relying on a single ensemble model, we employed the two best model combinations (C1 and C2) to enhance robustness. The observed differences in the average and maximum rankings of chemical classes between the two ensemble models (Figures 4B,C) suggest that the chemical space–activity relationship is represented differently in each model. This finding supports the strategy of combining both ensemble models during virtual screening to improve candidate molecule selection and decision-making processes.

The first noteworthy observation is that several of the molecules with the highest predicted probabilities in the C1 and C2 models are either well-known anticancer agents or structurally similar to such drugs. This result, independently, strengthens the reliability of the virtual screening approach but also indicates that some of the screened drugs could be acting as anticancer drugs but not necessarily specific to gastric cancer. For example, orsaponin and paclitaxel (Figure 3) have well-documented anticancer properties. Orsaponin (OSW-1) is a saponin isolated from *Ornithogalum saundersiae* that has demonstrated the ability to induce apoptosis in cancer cells in both *in vitro* and *in vivo* (xenograft) models (Zhang et al., 2017; Zhan et al., 2021). Although OSW-1 has been evaluated against several cancer types, such as colorectal and breast cancer, to our knowledge, it has not yet been studied in the context of gastric cancer or their cell lines. In contrast, paclitaxel is a broad-spectrum anticancer drug (Sharifi-Rad et al., 2021) that is also used in gastric cancer treatment, where it has been shown to improve overall survival (Fountzilas et al., 2024). In our database, paclitaxel was identified in *Corylus avellana*, *Taxus baccata*, *Taxus wallichiana*, *Taxus canadensis*, and other species of the *Taxus* genus. The detection of paclitaxel in *Corylus avellana* offers promising alternatives for biotechnological production through the cell culture of this species (Farhadi et al., 2020). Interestingly, *Corylus avellana* nuts are widely used in food products, and several studies, including clinical trials, have suggested a positive effect of these products in reducing the risk of esophageal and gastric adenocarcinomas (Hashemian et al., 2017; Cao et al., 2023). Molecules M5 and M6 (7-epi-10-Deacetyl Cephalomannine) are structurally related to paclitaxel (as taxane diterpenoids), but no previous studies were found specifically addressing their bioactivity. A similar situation applies to the other top-ranked molecules selected by the C1 and C2 models and presented in Figure 3.

Among the 340 molecules with predicted probabilities greater than 0.55, the most abundant subclasses are taxane diterpenoids (diterpenoids), flavanones (flavonoids), isoflavanones (isoflavonoids), agarofuran and daucane sesquiterpenoids (sesquiterpenoids), dimeric phloroglucinols (phloroglucinols), various subclasses of tryptophan alkaloids, limonoids (triterpenoids), and plant xanthones (xanthones class) (Figure 4A). However, when considering the predicted probabilities, the dominant groups are steroids, tryptophan alkaloids, sesquiterpenoids, phloroglucinols, and isoflavonoids (Figures 4B,C). This trend is also reflected in the predominant genera identified in Figures 5A,B. Genera such as *Taxus*, *Elaphoglossum*, *Glycyrrhiza*, *Seseli*, along with specific species like *Turraea obtusifolia* and *Melicope durifolia*, appear highly relevant not only for phytotherapy but also for the discovery of new bioactive molecules targeting gastric cancer.

As previously mentioned, the genera *Taxus*, *Glycyrrhiza*, *Elaphoglossum*, and *Seseli* comprise groups of diterpenoids, isoflavonoids, phloroglucinols, and tryptophan alkaloids, many of which correspond to the chemical classes with the highest predicted probabilities. Among the isoflavonoids predicted at the top by the C2 model, we identified 2′,4′,7-trihydroxy-5-methoxy-3′,6-diprenylisoflavan, glycybenzofuran, glyurallin A, and 4-[4-hydroxy-6-methoxy-5-(3-methylbut-2-enyl)-1-benzofuran-2-yl]benzene-1,3-diol. All of these compounds are found within the Glycyrrhiza genus, and some have documented anticancer properties (Ito et al., 2020; Wu et al., 2022). Indeed, flavonoids (particularly flavanones) and isoflavonoids from various *Glycyrrhiza* species have been widely regarded as key contributors to the anticancer activity associated with these plants (Jain et al., 2022; Frattaruolo et al., 2024). In the phloroglucinol group, compounds with high predicted probabilities (especially from the C2 model) were predominantly identified in *Elaphoglossum*, including elaphopilosins A, B, and D, along with structurally related molecules. To our knowledge, these specific elaphopilosins have not yet been directly associated with anticancer activity; however, related phloroglucinols and extracts from the same genus have demonstrated inhibitory effects against several cancer cell lines (Arvizu-Espinosa et al., 2019). Taxane diterpenoids, as previously discussed, were strongly favored by both C1 and C2 models, and are abundant in *Taxus* species. In the case of *Seseli*, published evidence supports the anticancer activity of several species, although most studies have focused on essential oils (Cinar et al., 2020; Chen et al., 2024; Vaglica et al., 2024) or coumarins (Zengin et al., 2021; Onder et al., 2023), with no specific tryptophan alkaloids identified as responsible for the observed effects (Zengin et al., 2021). In our study, the compounds identified in *Seseli* belong to the tryptophan alkaloid class. Although tryptophan alkaloids are known to have pro-apoptotic effects in cancer cells (Guo et al., 2022), we did not find specific reports linking the particular molecule identified in Seseli species to anticancer activity.

Unfortunately, the molecules belonging to the steroid group (Figure 3, M1) and the tryptophan alkaloids (Figure 3, M2, M4, and M7) do not have any specific reports of biological activity. Among the plant groups identified, the genera *Elaphoglossum* and *Seseli* show the least available evidence regarding potential anticancer effects, both at the plant level and for the molecules identified (elaphopilosins and certain tryptophan alkaloids). A closer examination of our results also highlights additional plants and molecules that could serve as promising candidates for future experimental anticancer screening efforts.

## 5 Limitations and future perspectives

The integration of *in silico* ensemble-based modeling with natural product databases offers a powerful approach for accelerating the discovery of novel bioactive compounds against gastric cancer. However, several critical steps remain necessary to translate these computational predictions into therapeutic advances. First, we can´t be sure that these molecules will show specific activity to gastric cancer cell lines. It could be possible to display a wide anticarcinogenic effect not only focused on gastric cells and even normal vs. pathological cells. In the future, the inclusion of other cell lines could open the possibility to explore these specificities. Second, *in vitro* validation of the prioritized molecules, particularly those from underexplored genera (such as *Elaphoglossum* and *Seseli*), is essential to confirm their anticancer potential and to elucidate their mechanisms of action. Parallel assessment of cytotoxicity against normal gastric epithelial cells will be crucial to identify compounds with favorable therapeutic windows. Third, the incorporation of toxicity prediction models and absorption, distribution, metabolism, excretion, and toxicity (ADMET) profiling into future virtual screening pipelines will enhance the reliability and safety of selected candidates. Expanding the modeling framework to include additional gastric cancer subtypes and drug resistance models could further refine compound selection and clinical relevance. Moreover, future studies could explore the synergistic effects of compound mixtures, particularly from the same plant source, reflecting the complexity of natural extracts traditionally used in phytotherapy. Integration of multi-omics data (e.g., transcriptomics and proteomics) and systems biology approaches may also provide deeper insights into the multi-target potential of selected natural compounds. Ultimately, the combination of computational, experimental, and systems biology methodologies will be critical to fully exploit the therapeutic potential of plant-derived molecules, offering promising new strategies for gastric cancer prevention and treatment.

## 6 Conclusions

In this study, we developed and validated two ensemble-based predictive models targeting the inhibitory effects of natural compounds on four gastric cancer cell lines: AGS, NCI-N87, BGC-823, and SNU-16. The models achieved robust predictive performance and significantly enhanced the identification of bioactive molecules 12–15 times greater than random selection. Virtual screening of over 100,000 natural compounds from 21,665 plant species revealed both known anticancer agents (e.g., paclitaxel and orsaponin) and novel candidates belonging to underexplored chemical classes such as phloroglucinols and tryptophan alkaloids. The genera *Taxus*, *Glycyrrhiza*, *Elaphoglossum*, and *Seseli* emerged as promising botanical sources. While some have established pharmacological profiles, others represent untapped resources with limited or no prior evidence of anticancer activity. These findings highlight the potential of phenotypic *in silico* screening for uncovering multifunctional compounds of natural origin. Further experimental validation, including cytotoxicity assays and mechanistic studies, is essential. Moreover, the inclusion of predicting models related to ADMET and cells selectivity could improve these predictions and advance the discovery of safe and effective plant-derived agents for gastric cancer therapy.

## Data Availability

The original contributions presented in the study are included in the article/Supplementary Material, further inquiries can be directed to the corresponding authors.
