# Supplementary material for: Discovering molecules and plants with potential activity against gastric cancer: an in silico ensemble-based modeling analysis
Source: Front Bioinform. 2025 Sep 30;5:1642039. doi: 10.3389/fbinf.2025.1642039 (PMC12518311; doi:10.3389/fbinf.2025.1642039)
Supplement: Supplementary file 2 [file Table1.docx]

**Supplementary Materials 2**

Table S1. Complete performance metrics computed for each model

|  |  | **GA Results** | | **BEST MODEL** | | | | | | | | | | |
| --- | --- | --- | --- | --- | --- | --- | --- | --- | --- | --- | --- | --- | --- | --- |
|  |  | **TEST** | | **TEST** | | | | | **EXTERNAL** | | | | | |
| **Cell Lines** | **Models** | **Mean**  **ACC** | **Mean**  **BCR** | **ACC** | **BCR** | **F1** | **SP** | **SE** | **ACC** | **BCR** | **F1** | **SP** | **SE** | **NV^1^** |
| SNU-16 | RF | 0.825 | 0.806 | 0.857 | 0.776 | 0.864 | 0.905 | 0.810 | 0.810 | 0.793 | 0.778 | 0.824 | 0.800 | 19 |
|  | DTREE | 0.796 | 0.778 | 0.786 | 0.748 | 0.791 | 0.810 | 0.762 | 0.857 | 0.825 | 0.833 | 0.882 | 0.840 | 26 |
|  | KNN | 0.834 | 0.814 | 0.810 | 0.810 | 0.810 | 0.810 | 0.810 | 0.810 | 0.793 | 0.778 | 0.824 | 0.800 | 17 |
|  | ENSEMBLES | 0.861 | 0.857 | 0.857 | 0.857 | 0.857 | 0.857 | 0.857 | 0.762 | 0.759 | 0.722 | 0.765 | 0.760 | 6 |
| NCI-N87 | RF | 0.847 | 0.822 | 0.816 | 0.802 | 0.809 | 0.826 | 0.808 | 0.816 | 0.802 | 0.824 | 0.808 | 0.826 | 23 |
|  | DTREE | 0.840 | 0.816 | 0.816 | 0.802 | 0.809 | 0.826 | 0.808 | 0.837 | 0.819 | 0.846 | 0.846 | 0.826 | 36 |
|  | KNN | 0.812 | 0.787 | 0.816 | 0.802 | 0.809 | 0.826 | 0.808 | 0.837 | 0.819 | 0.846 | 0.846 | 0.826 | 25 |
|  | ENSEMBLES | 0.929 | 0.915 | 0.918 | 0.909 | 0.913 | 0.913 | 0.923 | 0.857 | 0.838 | 0.863 | 0.846 | 0.870 | 11 |
| BGC-827 | RF | 0.688 | 0.667 | 0.703 | 0.687 | 0.703 | 0.714 | 0.692 | 0.709 | 0.705 | 0.689 | 0.706 | 0.712 | 17 |
|  | DTREE | 0.786 | 0.774 | 0.789 | 0.764 | 0.790 | 0.805 | 0.774 | 0.802 | 0.795 | 0.786 | 0.797 | 0.806 | 42 |
|  | KNN | 0.788 | 0.775 | 0.792 | 0.782 | 0.791 | 0.799 | 0.786 | 0.805 | 0.782 | 0.787 | 0.790 | 0.818 | 33 |
|  | ENSEMBLES | 0.858 | 0.851 | 0.863 | 0.853 | 0.860 | 0.857 | 0.868 | 0.815 | 0.809 | 0.800 | 0.811 | 0.818 | 15 |
| AGS | RF | 0.749 | 0.736 | 0.759 | 0.740 | 0.763 | 0.772 | 0.747 | 0.772 | 0.772 | 0.772 | 0.772 | 0.772 | 25 |
|  | DTREE | 0.768 | 0.753 | 0.766 | 0.737 | 0.761 | 0.747 | 0.785 | 0.778 | 0.769 | 0.777 | 0.772 | 0.785 | 23 |
|  | KNN | 0.764 | 0.749 | 0.753 | 0.744 | 0.755 | 0.759 | 0.747 | 0.778 | 0.769 | 0.780 | 0.785 | 0.772 | 36 |
|  | ENSEMBLES | 0.856 | 0.845 | 0.880 | 0.869 | 0.879 | 0.873 | 0.886 | 0.823 | 0.823 | 0.823 | 0.823 | 0.823 | 8 |

Notes. 1) (NV) Number of variables included in the models. In the case of ensembles this number refers to the number of models included.


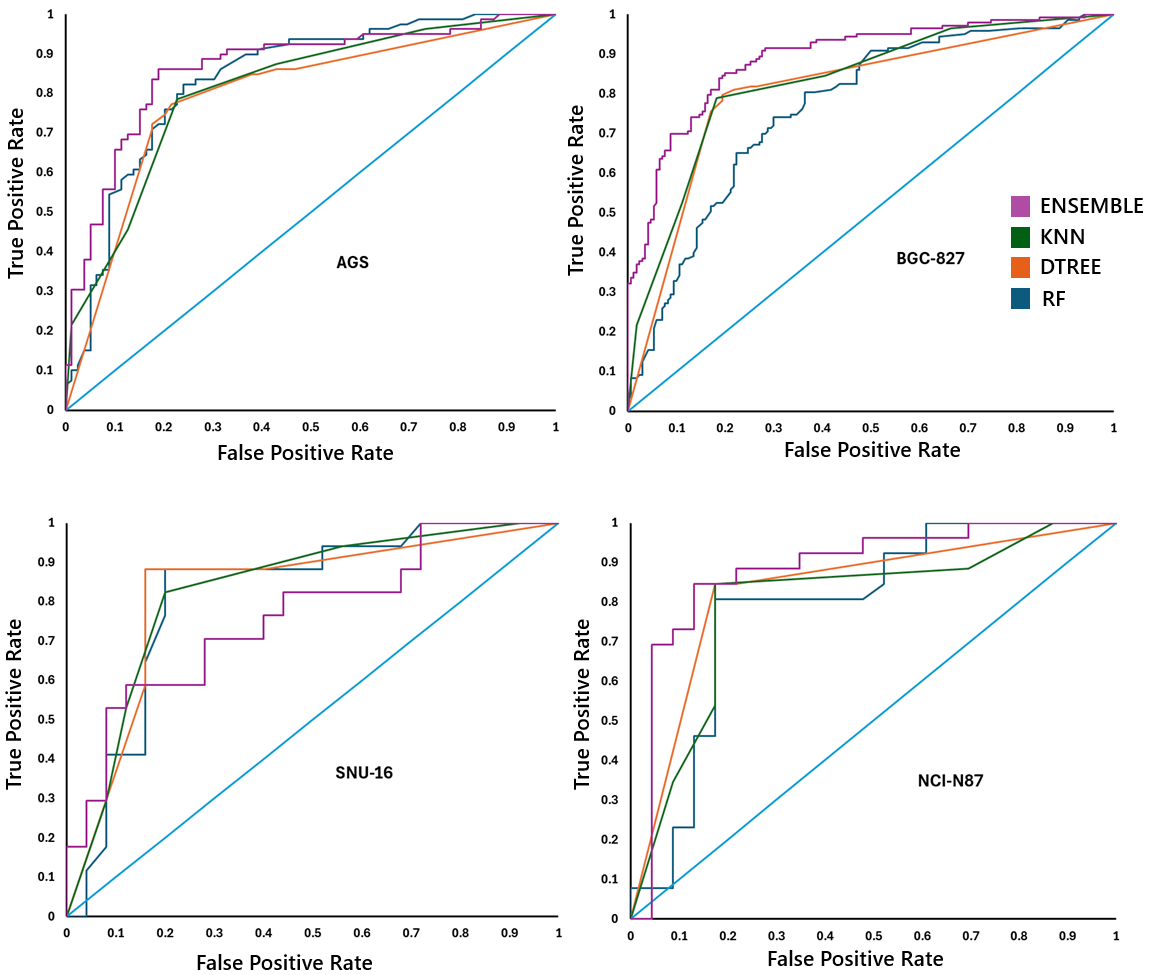


Figure S2.1. ROC-curve for each of the cell lines and the corresponding models including the ensemble.

Table S2. Complete performance metrics computed virtual screening models. Only the top 5 models are presented.

|  | **Selected models by cell lines** | | | |  | | | |
| --- | --- | --- | --- | --- | --- | --- | --- | --- |
|  | **SNU-16** | **NCI-N87** | **GBC-827** | **AGS** | **BEDROC**  **(α=160)** | **BEDROC**  **(α=180)** | **EF**  **(1%)** | **EF**  **(0.5%)** |
| C1 | ENSEMBLE | ENSEMBLE | ENSEMBLE | RF | 0.241 | 0.251 | 11.46 | 16.05 |
| C2 | ENSEMBLE | ENSEMBLE | ENSEMBLE | ENSEMBLE | 0.233 | 0.240 | 12.61 | 16.05 |
| C3 | ENSEMBLE | DTREE | ENSEMBLE | RF | 0.220 | 0.226 | 12.61 | 16.05 |
| C4 | KNN | ENSEMBLE | ENSEMBLE | RF | 0.230 | 0.238 | 10.32 | 13.76 |
| C5 | KNN | KNN | ENSEMBLE | ENSEMBLE | 0.223 | 0.228 | 11.46 | 13.76 |
